# Advances in physiological and clinical relevance of hiPSC-derived brain models for precision medicine pipelines

**DOI:** 10.3389/fncel.2024.1478572

**Published:** 2025-01-06

**Authors:** Negin Imani Farahani, Lisa Lin, Shama Nazir, Alireza Naderi, Leanne Rokos, Anthony Randal McIntosh, Lisa M. Julian

**Affiliations:** ^1^Department of Molecular Biology and Biochemistry, Simon Fraser University, Burnaby, BC, Canada; ^2^Centre for Cell Biology, Development, and Disease, Simon Fraser University, Burnaby, BC, Canada; ^3^Institute for Neuroscience and Neurotechnology, Simon Fraser University, Burnaby, BC, Canada; ^4^Department of Biological Sciences, Simon Fraser University, Burnaby, BC, Canada; ^5^Department of Biomedical Physiology and Kinesiology, Simon Fraser University, Burnaby, BC, Canada; ^6^Rotman Research Institute, Baycrest Health Sciences, University of Toronto, Toronto, ON, Canada

**Keywords:** precision medicine, hiPSCs, brain disorders, biomarkers, generative AI, brain organoids, neurophysiology

## Abstract

Precision, or personalized, medicine aims to stratify patients based on variable pathogenic signatures to optimize the effectiveness of disease prevention and treatment. This approach is favorable in the context of brain disorders, which are often heterogeneous in their pathophysiological features, patterns of disease progression and treatment response, resulting in limited therapeutic standard-of-care. Here we highlight the transformative role that human induced pluripotent stem cell (hiPSC)-derived neural models are poised to play in advancing precision medicine for brain disorders, particularly emerging innovations that improve the relevance of hiPSC models to human physiology. hiPSCs derived from accessible patient somatic cells can produce various neural cell types and tissues; current efforts to increase the complexity of these models, incorporating region-specific neural tissues and non-neural cell types of the brain microenvironment, are providing increasingly relevant insights into human-specific neurobiology. Continued advances in tissue engineering combined with innovations in genomics, high-throughput screening and imaging strengthen the physiological relevance of hiPSC models and thus their ability to uncover disease mechanisms, therapeutic vulnerabilities, and tissue and fluid-based biomarkers that will have real impact on neurological disease treatment. True physiological understanding, however, necessitates integration of hiPSC-neural models with patient biophysical data, including quantitative neuroimaging representations. We discuss recent innovations in cellular neuroscience that can provide these direct connections through generative AI modeling. Our focus is to highlight the great potential of synergy between these emerging innovations to pave the way for personalized medicine becoming a viable option for patients suffering from neuropathologies, particularly rare epileptic and neurodegenerative disorders.

## 1 Introduction

Precision medicine is a clinical approach that strives to develop targeted treatment paradigms for individual or specific groups of patients based on their unique genetic, molecular, physiological, environmental, and behavioral signatures (Kosorok and Laber, 2019). The promise of human induced pluripotent stem cells (hiPSCs) (Takahashi and Yamanaka, 2006; Takahashi et al., 2007) to play a central role in precision medicine approaches for the diagnosis and treatment of human diseases has been evident since their initial discovery, given that they reflect the full genomic complement of the patient from which they were derived (Agarwal et al., 2008; Gunaseeli et al., 2010; Marchetto et al., 2010; Chun et al., 2011). The synergistic emergence of advanced genome, tissue engineering, and high-throughput analytical approaches, combined with increased clinical accessibility and depth of genome sequencing technologies, is helping to make the promise of hiPSCs as central agents of precision medicine a reality. These advancing technologies allow researchers to model complex neurodevelopmental processes *in vitro* at cellular and molecular levels and to identify potential pathogenic mechanisms, biomarkers and therapeutic vulnerabilities, all in a patient-specific context. In under two decades (Takahashi and Yamanaka, 2006; Takahashi et al., 2007), hiPSCs have transformed our understanding of human development in both normal and disease contexts and new advances continue to mount at a rapid rate.

hiPSC-based technologies have been particularly impactful for the brain. Given the inaccessibility and scarcity of *ex vivo* human brain tissues available for study, and a relative lack of congruence between humans and animal models (Jucker, 2010; Knock and Julian, 2021), mechanistic investigation of the human brain has historically been challenging. hiPSCs now provide researchers with an invaluable tool to simply produce human neural cells and ever more complex tissues “in a dish.” Produced from the nuclear reprogramming of highly accessible sources of somatic cells, hiPSCs can be derived from any individual and strategies to establish these cells are becoming increasingly accessible (Liu et al., 2020). The initial somatic cell source used for hiPSC derivation was dermal fibroblasts obtained by punch biopsy of the donor’s skin; however, blood plasma and even urine samples are now more commonly used, making the process simpler and less invasive for donors and clinicians (Sohn et al., 2012). The reprogramming of somatic cells involves the introduction into the donor cells of four transcription factors–OCT4, SOX2, KLF4, and c-MYC–by either nucleofection or, more commonly due to increased efficiency, transduction with viral vectors (Takahashi et al., 2007). As hiPSCs have the same genotype as their somatic cell source obtained from the human donor, they typically carry disease-associated mutations of interest as well as other genetic variants specific to that individual. Isogenic hiPSC lines produced by genomic engineering to repair a disease-causing variant, or hiPSCs obtained from family members with a similar, though typically not identical, germline signature, serve as valuable controls for mechanistic investigations (An et al., 2012; Fujimori et al., 2018; Laperle et al., 2020). These standard methods allow researchers to study the unique impacts of inherited patient-specific mutations in the multiple cell types that can be differentiated from hiPSCs. Although cells and tissues derived from human stem cells cannot, on their own, provide information across all physiological scales, they can illuminate the genetic and molecular features of neurological disorders. With ongoing advances, such as the ability to produce a myriad of neural cell types, more complex region-specific three-dimensional (3D) organoid tissues, and non-neural cell types that are important contributors in the neural niche (Muffat et al., 2016; Abud et al., 2017; Centeno et al., 2018; Wörsdörfer et al., 2019), the mechanisms and causative factors identified will become more likely to serve as valid biomarkers or therapeutic targets. Moreover, the subtle differences in genetic make-up of these cultures makes them the perfect tool to characterize the development and progression of neurological disorders for each patient or specific classes of patients (such as those that share a common rare disease diagnosis).

A wide variety of protocols to generate neural cells and 3D tissues from hiPSCs have been developed (Muratore et al., 2014; Galiakberova and Dashinimaev, 2020; Mayhew and Singhania, 2023), and these vary in their complexity and therefore their degree of fidelity to the molecular, structural and functional heterogeneity of *in vivo* human brain tissue. These engineered models of the human brain are now routinely used to investigate molecular processes that underly key stages of brain development including stem cell maintenance and fate decisions, neuro- and glio-genesis, and neuronal network formation and synaptic properties (Liu and Zhang, 2011). hiPSC models are also increasingly used to elucidate mechanisms of not only neurodevelopmental but also aging-related diseases (Yagi et al., 2011; Stern et al., 2018; Knock and Julian, 2021). In parallel, methods to precisely modify the genome of hiPSCs, for instance to insert or repair disease-relevant mutations or lineage-tracing markers, and to produce neural cells and tissues *in vitro* that reflect the complexity of the native environment, have become increasingly sophisticated. Continued innovation in these areas will greatly enrich our ability to uncover both common mechanisms of disease between distinct disorders and to then determine how each patient population, or individuals within these populations, are unique–a central requirement to achieve precision-level medical care.

**FIGURE 1:**
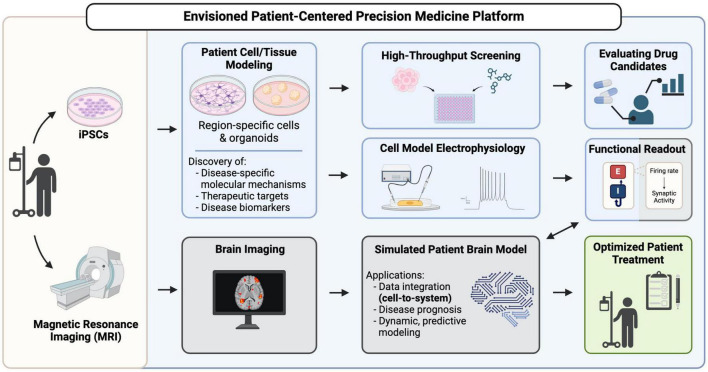
A vision for hiPSCs as key contributors in patient-centered precision medicine platforms. Patient-derived iPSCs are generated from patient somatic cells and are differentiated into brain region-specific cells and organoids for profiling, uncovering disease mechanisms and potential therapeutic targets. High-throughput screening using these patient-derived cells can reveal drug candidates that can go on for further evaluation of their efficacy. Functional readouts from electrophysiological assays, such as excitatory/inhibitory (E/I) balance in different neuronal populations can be integrated with brain imaging data in a simulated patient brain model to ultimately generate a predictive model to inform decision-making in patient diagnosis, prognosis and treatment.

As summarized in Figure 1, this review will highlight emerging technologies that together have great potential to center hiPSCs as powerful tools for physiological-level modeling of patient- and disease-specific pathological trajectories, and as agents for drug discovery, in neurological disorders. Efforts to consistently improve the depth and physiological relevance of high-throughput screening, neurophysiological analysis approaches, and the region-specific brain organoids and co-culture models (e.g., multi-region neural organoid models or those incorporating non-neural cell types) that can be established will have a particularly profound impact. We posit that a future exists in which hiPSCs are centered as agents to adequately inform clinical understanding and likely treatment trajectories for patients with genetic neurological disorders. However, to realize this goal the increasing repertoire of iPSC-derived human neural tissues, which elevate our ability to model the human brain with high complexity and fidelity, must continue to develop. Additionally, their integration with patient-derived biophysical data is needed. Personalization that spans cells to neural systems, achieved by integrating high resolution hiPSC-derived phenotyping data with multiscale biophysical models established from patient neuroimaging representations, can deepen our understanding of the underlying features of a patient’s disease and clarify complex mechanisms. Measuring cellular phenotypes across timescales, particularly after integration and validation with neurocognitive physiological parameters, may also uncover tissue and fluid-based biomarkers of bona fide disease trajectories and potential treatment approaches. These powerful emerging approaches and the critical advances that will follow from their integration, will transform the ability of researchers and clinicians to unravel the complexities of brain function and dysfunction and pave the way for personalized medicine becoming a viable and effective option for patients suffering from genetic neuropathologies.

## 2 A need for increased complexity to improve physiological relevance of hiPSC-based disease models

Human pluripotent stem cell-derived neuronal cultures and organoids have now been used to model numerous neurological disorders with a genetic basis. Early studies were largely focused on common diseases that impact development and aging trajectories like schizophrenia, bipolar disorder, autism syndromes, Alzheimer’s disease (AD) and Parkinson’s disease (PD) (Murai et al., 2016; Ochalek et al., 2017; Lewis et al., 2019; Bame et al., 2020; Laperle et al., 2020). Though more recently, rare genetic diseases have been increasingly represented in hiPSC disease modeling efforts and this focus promises to be especially impactful, both for our understanding of neuropathology in general and for our ability to effectively treat marginalized patient populations (Hosoya et al., 2017; Saito et al., 2018). Rare diseases frequently impact the brain, are typically genetic, and individually affect a small proportion of the human population (each fewer than 1 in 2000 live births) (Umair and Waqas, 2023). Given the large number of rare diseases that are thought to exist (an estimated ∼8000) (Somanadhan et al., 2023) and the small number of patients available for study in each case, there is currently little to no mechanistic understanding for most rare conditions. This reality precludes accurate diagnoses, limits options for effective treatment, and results in a lack of fundamental knowledge about how many genetic disorders impact the form and function of our brains. Though rapid advances in genetic testing are increasingly improving the odds of identifying causative genetic variants for distinct rare conditions, which can then lead to cell and molecular-level mechanistic understanding, this is not always successful. Furthermore, there is often a lack of adequate functional information to validate candidate mutations or determine which gene variant, sometimes among multiple potential candidates, is causing a patient’s disease.

Patient-derived hiPSC-neural models (paired with isogenic or familial controls) have already revealed important mechanistic details, and in some cases have elucidated therapeutic vulnerabilities, for many genetic disorders (Paşca et al., 2011; Liu et al., 2013; Barak et al., 2022). Few, however, have led to true clinical translation, and for most rare diseases hiPSC-derived models have yet to be developed at all. hiPSC approaches to model neural diseases have so far been fragmented, where investigations of pathology from individual groups typically emphasize specific aspects of a disease–such as cellular, physiological, cognitive, or clinical presentation–but lack an integrated view across multiple scales. Additionally, hiPSC-based studies typically assess individual cell types or a single brain region at a time, even though many neurological diseases are multifactorial involving contributions from different cell types and brain regions (Allen and Lyons, 2018; Fessel, 2023). This piecemeal strategy is inadequate to fully model and interrogate genetic brain diseases, as they are typically complex and impact multiple scales of brain form and function.

Establishing stem cell-based models for every rare genetic condition of the brain is a tall order. Though easing the burden, it has been promising to see as more studies as reported that specific pathogenic mechanisms often overlap between different genetic conditions. Furthermore, these overlapping mechanisms are often also observed in more common disorders for which mechanistic understanding is more abundant, and center on organelle dysfunction, altered proteostasis, and changes in the balance of excitatory and inhibitory (E/I) neurophysiological parameters (Reddy, 2009; Maestú et al., 2021; Pradhan and Bellingham, 2021; Wingo et al., 2022). Precision medicine approaches harnessing hiPSC technologies may therefore be accelerated by efforts to first group patients together who share similar disease signatures, even if they span distinct conditions. After this initial stratification, unique disease- or patient-specific phenotypic differences can then be more easily identified. This approach could help to put many patients on a promising diagnostic or treatment path more quickly and could greatly streamline mechanistic and therapeutic discovery endeavors, including identification of patient groups for which clinically approved drugs can be repurposed.

Before hiPSC-based technologies can reliably support disease diagnosis, trajectory predictions and treatment validation in the clinic (Figure 1), new paths to increase the throughput, fidelity, complexity, and physiological relevance of the neural cells and tissues they can produce must continue to emerge. Efforts to consistently improve the depth and accuracy of high-throughput screening and neurophysiological analysis approaches, and the region-specific brain organoids and co-culture models (e.g., multi-region neural organoid models or those incorporating non-neural cell types) that can be established from hiPSCs, will have a particularly profound impact. Technical strategies to analyze the impact of gene variants on cell fate decisions and molecular phenotypes, particularly those that are amenable to fluorescent-based imaging (Murai et al., 2016; Ochalek et al., 2017; Yang et al., 2017), are now quite robust. However, neurophysiological understanding at the cellular level requires quantification of the synaptic activity inherent in these cultures. Direct integration of cellular-level data with patient-derived neuroimaging representations (Figure 1) can be achieved by pairing datasets whose E/I balance parameters are closely matched (Figure 1; Schirner et al., 2018). Thus, we will focus our discussion on integrative cells-to-neural systems modeling of neurological disorders that are characterized by epilepsy and neurodegeneration, which consistently display measurable alterations in synaptic E/I balance. Notably, many neurological conditions–both developmental and aging-related–are marked by epileptic activity or neural tissue degeneration and their underlying mechanisms have been extensively investigated in animal and hiPSC disease models (Solodkin et al., 2011; Falcon et al., 2016; Bavassano et al., 2017; Jirsa et al., 2017; Rousseaux et al., 2018; Hashemi et al., 2020; Hommersom et al., 2022; Schirner et al., 2022; Jirsa et al., 2023). Given the prominence of epilepsy and neurodegeneration among neurological conditions, we therefore predict that the integrated patient-centered modeling platform we envision (Figure 1) will ultimately be amenable to most genetic brain conditions.

### 2.1 Advances in patient-centered modeling of neurodegenerative disorders

Neurodegenerative diseases (NDDs) are a diverse group of complex conditions characterized by progressive and irreversible dysfunction of the brain due to the continuous loss of specific neuron populations that are susceptible to degeneration (Forman et al., 2004). Animal models of NDDs have enhanced our understanding of the molecular pathogenesis of diseases such as AD, PD, and Huntington’s disease (HD) (Savitt et al., 2006; Yang et al., 2017; Dawson et al., 2018), with the hope of facilitating the discovery of multiple points of therapeutic intervention (Zeiss, 2017). However, findings in model organisms are not always translatable as some NDD pathologies are unique to humans. For example, because AD does not occur naturally in rodents, mouse models require genetic modification to cause the overexpression of human amyloid precursor protein (APP) and presenilin genes associated with familial AD (Sasaguri et al., 2017). The disease also manifests differently than in humans, where although the mice will develop amyloid plaques like those found in patients, they do not exhibit tauopathy or neurodegeneration (Selkoe, 2001). Due to the complexity and heterogeneity of NDDs in humans, animal models cannot fully recapitulate all physiological aspects of these disorders. Thus, there is a significant need for the development of advanced model systems that can effectively address the limitations of current animal models to enhance our mechanistic understanding of neurodegenerative diseases.

To address these concerns, many researchers are increasingly using human-based neuronal models to study NDDs. The synergy of novel hiPSC technologies with developments in genome-editing and sequencing has allowed for the investigation of cellular mechanisms during NDD pathogenesis. hiPSCs can also allow for the study of neurological diseases that don’t have underlying mutations yet identified, as patient-derived hiPSCs retain the full genetic information of any germline and sometimes somatic mutations, providing researchers with a tool to also study sporadic diseases. In the case of Amyotrophic Lateral Sclerosis (ALS), a heterogenous motor neuron disease which causes the progressive degeneration of motor neurons in the spinal cord and brain (Cluskey and Ramsden, 2001), 90–95% of cases are sporadic (sALS) (Kiernan et al., 2011), meaning they are not inherited unlike familial ALS (fALS). Before the development of hiPSCs, it was difficult to establish useful models of sALS. Now, not only are researchers able to establish human cellular models of sALS, but they have also used them to identify a therapeutic agent, ropinirole, which showed protective effects in both their fALS and sALS cell models (Fujimori et al., 2018). This discovery led to phase 1/2a clinical trials in patients with sALS testing ropinirole (Morimoto et al., 2023), where ropinirole was shown to slow disease progression, resulting in slower functional decline when patients were treated with the drug earlier. While further efforts are required to uncover the precise mechanism of action for ropinirole in ALS, this body of work demonstrates that hiPSC culture models have the capability to be used in precision medicine pipelines to identify drug candidates and predict the responsiveness of patients to treatments (Farkhondeh et al., 2019; Okano and Morimoto, 2022).

The bulk of hiPSC-neural modeling studies to date have centered on models of the forebrain. However, it is well known that *in vivo*, NDDs often involve heterogenous representations across multiple brain regions. The forebrain may be involved in some pathological process, but it is not always the primary tissue region affected with other brain regions often displaying the first signs of the degeneration cascade. This means that many existing human cell models of NDD do not address the appropriate cell and tissue types. Recent innovations that are transforming our ability to produce region-specific brain organoids, particularly the cerebellum, are starting to fill this critical gap (Silva et al., 2020; Atamian et al., 2024). For example, the cerebellum is an important contributor in neural atrophy disorders such as Familial Ataxia Syndromes (Silva et al., 2020), Multiple System Atrophy (MSA) (Ciolli et al., 2014), and Creutzfeldt-Jakob Disease (CJD) (Zerr and Parchi, 2018). New findings suggest that cerebellar gray matter atrophy is also involved in many conditions previously attributed to the degeneration of the cerebral cortex such as AD, PD and Frontotemporal Dementia (FTD) (Guo et al., 2016; Gellersen et al., 2017; Seidel et al., 2017), suggesting that a more thorough investigation of the cerebellum can be insightful for human NDD modeling.

One such example of cerebellar atrophy disorders are the spinocerebellar ataxias (SCAs), a group of over 40 rare hereditary progressive movement disorders that primarily affect neurons in the hindbrain and cerebellum, with involvement in some forms in the spinal cord and cerebral cortex (Klockgether et al., 2019; Chirino-Pérez et al., 2021; Guo et al., 2023; Pilotto et al., 2023). To study this class of NDDs in a human model, researchers can use hiPSCs derived from both healthy individuals and SCA patients to generate cerebellar organoids, which can consistently reproduce multiple types of functional cerebellar neurons (Atamian et al., 2024). This very recent establishment of a functional human model of the cerebellum is an integral step to improving our understanding of human neurophysiology and pathology, as studies have shown that the human cerebellum differs drastically from that of rodents. For example, in contrast to mouse, the human cerebellum has more outer radial glia in an outer subventricular zone during development that drives human cerebellar expansion and gyrification (Nowakowski et al., 2016). Other differences in human cerebellum development are in rhombic lip (RL) morphology and the existence of substructure zones such as ventricular (RL*^VZ^*) and subventricular zones (RL*^SVZ^*), as well as internalization of the RL into the posterior lobule to form a tightly packed pool of cells (Haldipur et al., 2019). Differences in development ultimately translate to a different mature tissue, highlighting the importance of a human-based model to capture all aspects of neurophysiology and pathology in the cerebellum. While it can be argued that because hiPSC models elucidate mechanisms of disease in an earlier developmental model they might not be well suited for modeling diseases that arise later in adulthood, there is evidence to suggest that events during early prodromal stages in these developmental models can shed light on the acquisition of late-stage syndromic phenotypes (Caldwell et al., 2015; Tremlett and Marrie, 2021). This prodromal window may also be a more suitable therapeutic window to recover brain homeostasis.

The recent development of brainstem organoids (Eura et al., 2020; Lui et al., 2023), an area of extensive atrophy in many patients with SCA, will help to further develop appropriately complex models for this class of NDDs. Incorporating multiple brain region-specific organoids that are relevant to a given NDD–for example forebrain, cerebellum and brainstem–can permit analysis of degenerative mechanisms both within and between distinct affected regions. The rapid advance of brain organoid assembloid technologies (further detailed below), where unique types of organoids are physically connected to permit observation and assessment of neuronal network formation across brain regions, is substantially supporting efforts to improve physiological relevance in neurological disease models.

### 2.2 Advances in patient-centered epilepsy modeling

Epilepsy, a condition characterized by persistent predisposition to experiencing seizures, is thought to affect a staggering ∼1% of the population, or about 80 million people worldwide (Beghi, 2020). Epilepsy can arise due to one of many genetically defined conditions or, more often, as a secondary feature of other neuropathological conditions or from unknown causes. The exact mechanisms driving the underlying neural network dysfunction in these disorders largely remain to be uncovered (Perucca and Perucca, 2019), but *in vitro* modeling of inherited epilepsy disorders with hiPSCs has opened new doors for mechanistic investigations and drug discovery. The added value emerging from hiPSC-derived neural epilepsy models again highlights the limitations of existing animal models to capture certain human-specific features, which are critical to the establishment of altered brain architecture and synaptic networks that underlie these syndromes (Blair et al., 2018; Knock and Julian, 2021; Eichmuller et al., 2022). As with NDDs, in many epilepsies a genetic cause is presumed but the gene variant is unknown. hiPSC approaches to model brain development and neural network function from epileptic patients can therefore inform pathogenic mechanisms both for patients with well-defined mutations and for those whose genetic cause is not yet identified. In the latter case, the presence of epileptic phenotypes in hiPSC neural models derived from patients, compared to those from familial controls, can help to diagnose individuals by establishing if their syndrome does indeed have a genetic basis.

Many genetic epilepsies exist, but we will focus on a select few for which hiPSC models have provided significant advances–Dravet syndrome, a severe myoclonic epilepsy characterized by febrile seizures that begin in infancy, and the class of disorders termed malformations of cortical development (MCDs) (Chen et al., 2014; Costa et al., 2016; Sun and Dolmetsch, 2018; Majolo et al., 2019; Klofas et al., 2020). MCD describes numerous disorders that are characterized by altered brain development causing architectural and neuronal network abnormalities, which almost invariably lead to altered cognition and severe epilepsy that are typically treatment resistant (Oegema et al., 2020). Tuberous sclerosis, focal cortical dysplasia, and megalencephaly are among the most commonly studied MCDs, and hiPSC-derived *in vitro* models for these conditions continue to improve in their fidelity and capacity to model disease-relevant features (Li et al., 2017; Avansini et al., 2022; Eichmuller et al., 2022).

Given the relative ease of electrophysiological analyses in pure neuronal cultures, hiPSC-based models of epilepsy have predominantly focused on the derivation and functional assessment of 2D cultures of neurons, sometimes also including astrocytes. Typically, these are telencephalic forebrain neurons given the propensity of basic neuronal differentiation protocols to yield forebrain cells (Muratore et al., 2014). Efforts to model Dravet syndrome with patient-derived hiPSCs, which carry a deleterious mutation in the Nav1.1 sodium channel, indicate that cultures of telencephalic interneurons carrying this variant have reduced sodium current density and action potential output; however, no phenotypic change was observed in excitatory neurons (Sun et al., 2016). These results are in line with previous findings in a mouse model of Dravet syndrome (Yu et al., 2006) and highlight differences in the contribution of distinct neuronal subtypes to the epileptic state in Dravet patients. Although only in 2D culture models, these results showcase the potential of hiPSCs to translate cellular-level phenotypes to clinical presentation, and further suggest the potential to evaluate the efficacy of potential therapeutic compounds at the cellular level. hiPSC models have similarly been used to shed light on the antiepileptic properties of cannabidiol (CBD) in Dravet syndrome and to study its mechanism of action. Researchers administered CBD to telencephalic neurons produced from patient-derived hiPSCs, which increased the excitability of inhibitory neurons and decreased the excitability of excitatory neurons, without altering sodium channel currents in these cell types (Sun and Dolmetsch, 2018). These observations indicate that the effect of CBD is also targeted to specific neuronal subtypes and is independent of sodium channel activity.

MCDs stand as the leading cause of medically refractory epilepsy in children (Oegema et al., 2020). In adults, the MCD Focal Cortical Dysplasia ranks as the third most common cause of medically intractable seizures (Kabat and Król, 2012). Molecular analysis of neurons from patients with Focal Cortical Dysplasia Type II identified dysregulation of several genes associated with neuronal migration during neurogenesis and embryonic neural progenitor cell differentiation, which is a key factor that underlies development of the malformed cortical tissue within the brains of these patients that underlies their epileptic activity (Majolo et al., 2019). hiPSCs carrying loss-of-function mutations in *DEPDC5*, encoding a GATOR1 complex member, show overactivation of the mammalian target of rapamycin complex 1 (mTORC1) signaling node, resulting in epileptic episodes which can be alleviated by administering the mTORC1 inhibitor rapamycin (Klofas et al., 2020). A growing number of studies indicate that mTORC1 plays a significant role in many neurological diseases due to its involvement in autophagy, proteostasis, cell proliferation and migration, and its interactions with multiple signaling pathways that modify the cell’s energetic state, neurotransmitters, and growth factors (Cuyàs et al., 2014; Guo et al., 2018).

mTORC1 disruption is heavily implicated in multiple forms of MCDs, including megalencephaly disorders and tuberous sclerosis (TS) which is caused by mutations in the *TSC1* or *TSC2* genes (Hernandez et al., 2007; Kashii et al., 2023), leading to persistent mTORC1 hyperactivation. Disruption of the mTORC1 pathway leads to formation of benign tumors and generalized tissue malformations in multiple organs including the brain (Delaney et al., 2014; Crino, 2020; Delaney et al., 2020; Dhaliwal et al., 2024). Epilepsy manifests in up to 90% of TS patients (Rocktäschel et al., 2019), which is due to the presence of these low grade architecturally abnormal tumors or “cortical tubers” (Delaney et al., 2014; Frost and Hulbert, 2015). Patients with TS inherit a mutation in one allele of *TSC1* or *TSC2* and subsequent loss of heterozygosity in a subset of cells gives rise to focal regions of malformed brain tissue. Complete loss of *TSC1* or *TSC2* is therefore required to observe the full spectrum of disease-relevant phenotypes. Mouse models are not fully sufficient to investigate the molecular mechanisms of tuber formation, since homozygous germline *TSC1* or *TSC2* mutants are embryonic lethal due to failure of neural tube closure (Kobayashi et al., 1999; Møller et al., 2016), and various conditional knockouts fail to recapitulate the architecture of cortical tubers. The approach of differentiating neurons and glia from human pluripotent stem cells has proven to be an ideal and unique tool to investigate how *TSC1* or *TSC2* loss impacts the development of neural precursors, neurons, and glia, and the neural networks that they form, in the human brain (Nadadhur et al., 2019; Delaney et al., 2020). Reports on hiPSC-derived neuronal cultures have shown that deletion of *TSC2* leads to structural abnormalities in neuroectodermal rosettes, reminiscent of the defective neural tube closure observed *in vivo* (Costa et al., 2016; Winden et al., 2019). More directly relevant to epileptic phenotypes, molecular, cellular, and electrophysiological characteristics of dysplastic neurons from cortical tubers induced by deletion of *TSC2* have been routinely measured in forebrain neuronal cultures and rescued by the mTORC1 inhibitor rapamycin, a derivative of the leading pharmaceutical treatment for TS (Costa et al., 2016; Nadadhur et al., 2019; Winden et al., 2019; Delaney et al., 2020). Despite these advances in 2D neuronal cultures, TS is a profound example of the importance of 3D organoid modeling to more accurately reflect human disease phenotypes, as only in cerebral organoids has the architecture of cortical tubers been recapitulated (Blair et al., 2018; Eichmuller et al., 2022). The synergy of these hiPSC-derived neural cultures with advances in the electrophysiological assays discussed in the following section make them an ideal tool to study the impacts of potential drug therapeutics on epilepsy phenotypes *in vitro*.

## 3 Advancing hiPSC neural circuitry measurements with region-specific brain organoids

Our understanding of the early stages of neural circuitry development in humans has long been impeded by a lack of access to fetal and neonatal brain tissue. Obvious ethical concerns surrounding the use of such tissues to directly observe and manipulate developing neural circuitry have precipitated the use of alternative animal models to uncover key insights into human brain development. Rodents are widely used due to their genetic tractability and well-characterized neuroanatomy (Glowinski and Iversen, 1966). Non-human primate models are also used, albeit more sparingly, as they are closer to humans in terms of brain complexity and behavior (Feng et al., 2020). Additionally, zebrafish have transparent embryos that allow visualization of live early neural development (Sakai et al., 2018), while fruit flies and *C. elegans* have a simple nervous system, providing a more manageable model for exploring neural circuits (Sengupta and Samuel, 2009; Bellen et al., 2010). While the use of animal models has been essential to understanding the basic nature of brain disorders that impact humans and continue to be an invaluable tool, they also come with their own disadvantages. Confounding factors such as captivity and housing conditions, which can cause stress and impacts on animal welfare, can negatively impact the validity and reproducibility of experimental results. It is also important to note that despite some similarities between humans and animal models, there is a lack of congruence to many features of the developing and adult human brain, which is thought to largely underlie the substantial issue of translational failure as promising therapeutic strategies are moved from experimental models to the clinical domain (Marshall et al., 2023).

The introduction of hiPSC-derived neural models, superseded by human embryonic stem cells (hESCs), in the last 20 years (Takahashi and Yamanaka, 2006; Marchetto et al., 2010; Cheung et al., 2011) has provided a profound tool to study neural circuitry in early human development, when in the past we have mostly been limited to postmortem tissues and neuroimaging data (Manzini et al., 2021). As a result, researchers are now able to use electrophysiological tools to assess the functional attributes of neurons in culture, including: (1) regular and high-density multi-electrode arrays (MEA) for prolonged, non-destructive recordings of electrical activity and network dynamics (Negri et al., 2020; Habibey et al., 2022); (2) patch clamping for direct measurement of neuronal activity (Bardy et al., 2016); (3) calcium imaging using fluorescent calcium indicators to monitor changes in intracellular calcium levels, serving as a proxy of neuronal activity (Estévez-Priego et al., 2023); (4) voltage-sensitive dye imaging using fluorescent dyes that change their fluorescent properties in response to changes in membrane potential (Glover et al., 2008); and (5) optogenetics, harnessing genetically engineered neurons that express light-sensitive proteins called opsins, permitting selective activation or inhibition of neurons by light stimulation to manipulate neuronal activity *in vitro* (Deisseroth et al., 2006). It is important to note that these 2D models lack native tissue architecture and complex intercellular interactions, which poses a challenge to studying neurodevelopment *in vitro* (Adlakha, 2023). The development of the 3D neural organoid culture method has provided a platform for the investigation of brain development in a more complex tissue environment. Many of the previously mentioned electrophysiological assays are now being applied to investigate neuronal function in 3D brain organoid models (Birey et al., 2017; Xiang et al., 2017; Fair et al., 2020; Tasnim and Liu, 2022; Yang et al., 2024). The cellular diversity of brain organoids allows researchers to study the diverse interactions that contribute to neural circuit function. Additionally, as these organoids can be maintained in culture for extended periods, including recent advances with organoid transplantation into mouse brain tissues to permit more advanced human neuronal cell and electrophysiological development (Revah et al., 2022; Kelley et al., 2024) researchers can study the development and maturation of neural circuit network dynamics over time (Fair et al., 2020). These 3D systems have been used to model disorders with known electrophysiological abnormalities, such as epilepsy (Hirose et al., 2020; Nieto-Estévez and Hsieh, 2020; Saberi et al., 2022), and can be further applied to substantiate therapeutic testing via high throughput drug screening applications.

**FIGURE 2:**
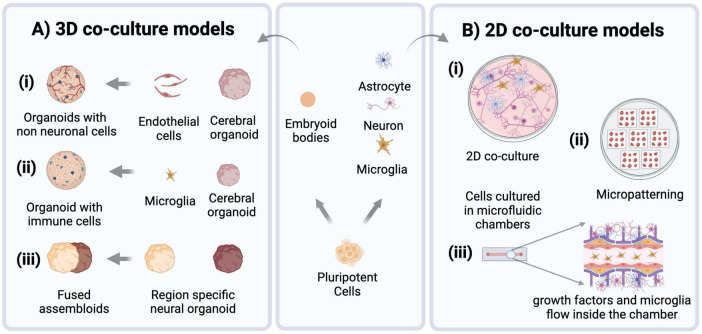
Modeling approaches for brain disorders using hiPSCs. Pluripotent stem cells are routinely used to derive embryoid bodies for heterogenous 3D organoid production, or cultures of neural and non-neural (e.g., microglia) cells that can then be combined to establish co-cultures of specified cell types (center panel). **(A)** 3D brain organoid models increasingly use co-culture approaches to enhance their physiological relevance by incorporating non-neural cell types such as (i) endothelial cells (ii) microglia, and (iii) generating region-specific organoids which can be fused to create assembloids. **(B)** 2D co-culture models: (i) culturing neurons, microglia, and astrocytes in a dish. (ii) Micropatterns, Cells are patterned into arrays of specific shapes and sizes to control their spatial organization. (iii) Microfluidic models of brain tissue are created to control the flow of nutrients and incorporate non-neural cell types through small scale channels.

To study the interactions between cells from different brain regions, researchers have developed co-culture methods to combine brain-region specific organoids into a single integrated model (Figure 2A; Bagley et al., 2017; Xiang et al., 2017). Since the introduction of these methods, there have been many multi-region organoid co-culture models generated to enable human neural circuitry studies in a dish to investigate complex neuropathophysiologies. The combination of these various neural organoids has allowed researchers to study key developmental processes in a more physiologically relevant model, such as interneuron migration and various neural circuitry projections. Table 1 lists some of these models that have been developed in recent years.

**TABLE 1:** Examples of multi-region organoids.

Multi-region organoid model	Aspect of neural circuitry studied	Disease modeled	Publications
Dorsal forebrain & ventral forebrain	Interneuron migration	Timothy syndrome	Bagley et al., 2017; Birey et al., 2017; Xiang et al., 2017; Sloan et al., 2018
Cortical + thalamic	Thalamo-cortical projections of ascending sensory input	Schizophrenia, bipolar disorder, autism spectrum disorders, epilepsy (Angulo Salavarria et al., 2023)	Xiang et al., 2019
Cortical + striatal	Projections of motor planning circuits	Phelan-McDermid syndrome	Miura et al., 2020
Cortical + spinal + skeletal muscle	Cortico-spinal projections	Amyotrophic lateral sclerosis, spinal muscular dystrophy	Andersen et al., 2020
Retina + cortical + thalamic	Projections of circuitry of the visual pathway	Ocular neurodegeneration	Fligor et al., 2021

Table showing the region-specific organoids necessary to generate the listed multi-region organoid models, including the aspects of neural circuitry and disease that can be investigated with the model, as well as publications that have used these models.

The electrophysiology data collected from these multi-region assembloid models allows for a detailed assessment of neural cell and circuit interactions between different brain regions, particularly as inhibitory interneurons integrate within excitatory neuron networks in the dorsal and ventral forebrain organoid co-culture model, a phenomenon that is highly reflective of neural circuit development in the human brain.

## 4 Advancing understanding of the brain microenvironment with co-culture of non-neural cell types

*In vitro* research on the human brain has primarily centered around neural cell types, including neurons, astrocytes and oligodendrocytes. However, when aiming to establish platforms to facilitate effective precision medicine strategies, which should mirror the *in vivo* condition as closely as possible, it is crucial to acknowledge the critical role that non-neural cells play in brain physiology and pathology. These cells can include pericytes and endothelial cells, which constitute the blood-brain-barrier. One of the most prominent non-neural cell types in the brain, which have emerged as particularly impactful regulatory cells across the lifespan, are brain-specific immune cells called microglia (Colonna and Butovsky, 2017; Mehl et al., 2022). During human brain development, microglia originate from primitive myeloid progenitors derived from the yolk sac. These progenitors migrate into the brain early during development, where they then differentiate into functional microglial cells. Microglia constitute approximately 5–16% of the total cell population in a fully developed brain and are maintained throughout life through self-renewing divisions (Masuda et al., 2020). As brain resident immune cells, microglia play a significant role in normal brain development and homeostasis. They are found to accumulate near dead neurons and are involved in phagocytosis of cell debris and degenerating axons. This role is more evident in a state of neural injury or stroke (Colonna and Butovsky, 2017). Microglia have also been associated with the stem cell niche in the brain at the subventricular zone and neocortex, where they regulate the differentiation of neural stem cells by selective cell engulfment (Xu et al., 2021). Moreover, they regulate the formation of neural circuits and maintain a balance of excitatory and inhibitory neurons by affecting inhibitory interneuron migration (Bohlen et al., 2019; Mehl et al., 2022). In addition to these widespread roles, microglia affect neuronal function through their crosstalk with other glial cells (Thion et al., 2018).

Microglia are morphologically dynamic, ranging from thin, ramified structures under normal physiological conditions and transitioning to an activated state and hypertrophic morphology in response to certain stimuli (such as injury, infection, or neuroinflammation). In the active state, they show increased proliferation while secreting inflammatory factors such as chemokines and cytokines, which profoundly affect brain function [reviewed in Bohlen et al. (2019)]. Their activation, therefore, impacts brain physiology at many levels including neural circuits, cell populations, synapses, and neurotransmitter signaling (Bohlen et al., 2019; Mehl et al., 2022). Considering the many key functions they carry out, it is no surprise that microglia have a significant role in brain disorders spanning psychiatric conditions, such as schizophrenia, bipolar disorder, and autism, to neurodegenerative diseases like AD, PD and ataxias (Willis et al., 2020; Silvin et al., 2022; Tagliatti et al., 2024). *In vitro* models that lack microglia often present clear limitations in their ability to fully elucidate the mechanisms underlying neurological disorders. Thus, integrating immune cells like microglia into human neural cell models is paramount, and overlooking their contribution can result in significant gaps in our understanding of complex regulatory mechanisms in brain development and disease pathogenesis (Ronaldson and Davis, 2020; du Chatinier et al., 2023).

For *ex vivo* studies, researchers typically isolate primary microglia from postmortem human, rodent or non-human primate brain tissue (Roqué and Costa, 2017). The use of primary microglia comes with certain limitations, including inconsistent phenotypes due to the positive disease status of the donor, altered activation states during the process of isolation, and, in the case of humans, lack of access to high-quality human postmortem tissue (Timmerman et al., 2018). Immortalized microglial cell lines, such as BV2 (derived from mouse), HMC3 (derived from human embryo), and CHME-5 (derived from adult human tissue) are also available (Dello Russo et al., 2018; Tian et al., 2019). While convenient, these cell lines show heterogeneity, genetic instability, and differences in differentiation state, which may impact their relevance to *in vivo* conditions (Das et al., 2016; Aktories et al., 2022). In addition to primary and immortalized microglia, protocols have been designed to differentiate or “induce” microglia (iMG) from embryonic or induced pluripotent stem cells (Speicher et al., 2019). iMG generated by these protocols resemble *in vivo* microglia in the context of their ability to phagocytose exogenous substances and respond to immune stimulation (Abud et al., 2017).

Microglia exhibit distinct responses to their microenvironment when cultured in 3D as opposed to traditional 2D cultures. In 3D environments, microglia display altered morphology, enhanced motility, and differential gene expression profiles compared to their 2D counterparts, reflecting the influence of spatial cues and cell-cell interactions on microglia behavior (Luchena et al., 2022). Therefore, 3D cultures with microglia (Figure 2A) can better recapitulate aspects of tissue architecture and cellular organization, providing a more accurate model for studying microglia behavior and function *in vitro* and their impacts on the developing brain. iMGs have been shown to incorporate into brain organoid tissue when co-cultured with them and show functional relevance to *in vivo* microglia post-integration (Abud et al., 2017). Xu et al. (2021) showed that iMG integrated into cerebral organoids engulf neural progenitor cells (NPCs), apoptotic cells, and neuronal synapses. iMGs when co-cultured with brain-specific organoids (i.e., with dorsal and ventral forebrain organoids), displayed different migration ability, intracellular Ca2 + signaling, and responses to pro-inflammatory stimuli (with higher expression of TNF-α and TREM2 in the ventral organoid-microglia group) (Song et al., 2019). Additionally, the transcriptome profile of iMGs cultured with dorsal brain organoids differs from that of ventral brain organoids, demonstrating that they are capable of responding to different neural niches *in vitro* (Hasselmann et al., 2019).

Microglia are also essential for proper development and function of the retina, and thus play a crucial role in the pathogenesis and progression of retinal disorders. The native state of retinal organoids is not captured accurately if microglia are absent. Therefore, to precisely represent the native retina, hiPSC-derived retinal organoids containing microglia are developed either by direct co-culture or by differentiating microglia in parallel within the organoids (Bartalska et al., 2022; Gao et al., 2022; Usui-Ouchi et al., 2023).

Microglia have also been incorporated into more defined engineered models to study their interaction with neural cells more precisely (Figure 2). These include microfluidic devices that allow dynamic control over microenvironmental factors and replication of *in vivo* nutrient gradients. Organ-on-a-chip models, which incorporate microfluidics to mimic physiological conditions of the brain and involve more intricate modeling of brain tissue, for example components of neural tissue juxtaposed with cells of the blood-brain-barrier (BBB) (Amirifar et al., 2022). Tissue micropatterning harnesses the power of growth restriction to pattern cells into self-assembled structures that reflect *in vivo* tissue organization, and interestingly microglia show structural diversity when grown on different micropatterned surfaces (Amadio et al., 2013). This approach can help to correlate the structural differences of microglia with their distinct functional identities. Co-culturing microglia with micropatterned neural tissue can reveal how microglia respond to different patterns of neural organization, providing insights into various subpopulations and their roles in development (Figure 2B). Simulation models further complement these approaches by enabling the computational study of microglial functions, such as their response to stress or inflammation and integrating that information into predictive models of brain function and disease. Ao et al. (2021) developed an organoid-on-chip model by synthesizing a device in a tubular shape where EBs are cultured to form a tubular organoid with an inner channel for the flow of nutrients and non-neural cells such as microglia, thus offering comprehensive insights into their functions and cellular interactions. Similarly, Pediaditakis et al. (2022) synthesized a microfluidic model to culture neurons, microglia, astrocytes and pericytes together, creating a neurovascular unit to study neuron and glia interactions in the brain microenvironment. Amadio et al. (2013) showed that microglia can move through microfluidic channels and travel up to 55 μm within 12 h. Culturing microglia on micropatterned surfaces and restricting their growth spatially can help to obtain more reproducible phenotypes. It has been shown that microglia possess distinct morphologies when cultured on different biomimetic micropatterned surfaces with different shapes correlating to diverse functional identities (Amadio et al., 2013). Finally, to achieve an integrated understanding of biological processes, computational models are also employed [as reviewed in Anderson and Vadigepalli (2016)]. Modeling studies demonstrate a strong correlation between microglial morphology and their function. The introduction of microglia and cytokine signaling into brain simulation models can further the knowledge of their plasticity and variability and the impact of different microglia states on brain form and function (Anderson and Vadigepalli, 2016). The neuroinflammatory environment can be created by co-culturing microglia with neurons and astrocytes, but to capture physiologically relevant microglial phenotypes more robust models must be created. These models should involve use of vasculature, endothelial cells and spatial cues to understand role of microglia in health and disease (Figure 2).

## 5 hiPSC-derived models as tools to identify disease-associated biomarkers

Disease-specific biomarkers have a crucial role in precision medicine, as they can help to identify specific characteristics that indicate the presence of progression of a particular disease, allowing clinicians to make accurate diagnoses, categorize patients into subtypes based on their biomarker signature and ultimately develop targeted interventions and treatments. Biomarkers can exist in the form of specific proteins, metabolites, genetic mutations, physiological parameters (e.g., blood pressure) or even brain imaging patterns that indicate a patient’s prognosis, in addition to the stage of disease pathogenesis and progression (Chen et al., 2011). Although the use of biomarkers in precision medicine has been mostly applied in oncological indications, such as estrogen receptor, progesterone receptor, and human epidermal growth factor receptor 2 (ER/PR/HER2) for breast cancer (Gamble et al., 2021), applications to a variety of target organs - including the brain - do exist.

Common neurodegenerative diseases such as AD affect millions of people worldwide and are especially prevalent clinically in the aging population. To make an accurate and differential diagnosis, a combination of structural and functional imaging, genetic, blood-based and cerebrospinal fluid (CSF) biomarkers are used by clinicians. In the case of AD, increased CSF levels of total-tau and phosphorylated-tau protein along with decreased levels of CSF Aβ1−42 create the typical AD biomarker profile found in most AD patients (Bouwman et al., 2022). While these CSF biomarkers represent the gold standard for the molecular characterization of neurodegenerative diseases, collecting CSF from patients for long-term observation is not feasible due to its invasive nature (Koníěková et al., 2022). While blood-based biomarkers exist for these neurological diseases, accurately measuring them poses a challenge as these brain-derived biomarkers are present at low concentrations in this fluid due to the blood-brain barrier (Zetterberg and Burnham, 2019). Additionally, certain biomarkers for AD may have potential interference from heterophilic antibodies and can be at risk of proteolytic degradation by proteases in blood plasma (Zetterberg and Burnham, 2019). Due to these considerations, an alternative source for brain-derived biomarkers is needed to enable further discovery and characterization for patients with rare neuropathophysiologies (Zetterberg and Burnham, 2019).

**FIGURE 3:**
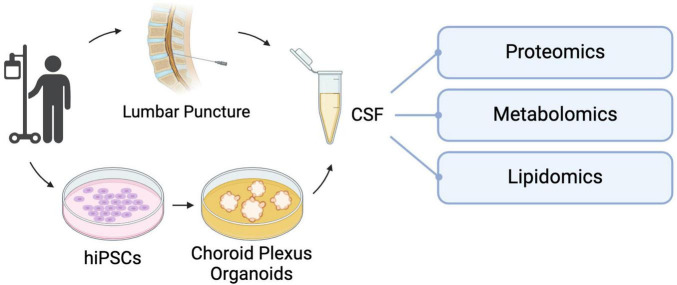
hiPSC-derived choroid plexus organoids present an alternative method of collecting CSF for biomarker analysis without the need for invasive lumbar punctures. This *in vitro* CSF-like fluid can be used for targeted investigation of factors of interest or unbiased analyses like proteomic, metabolomic and lipidomic screening to uncover unique molecular signatures that can be used to make differential diagnoses and track disease progression and prognosis.

A recent technology describing the generation of human barrier-forming choroid plexus (ChP) neural organoids capable of producing CSF-like fluid (Pellegrini et al., 2020) may present a potential solution to the challenges with CSF-based biomarkers. Organoids are defined as *in vitro* cellular models formed via self-organization, including multiple organ-specific cell types that demonstrate functional and cytoarchitectural traits associated with a specific organ (Paşca et al., 2022). Fitting this definition, ChP organoids demonstrate key features of the human ChP, forming tight barriers that selectively regulate the entry of small molecules, and secrete a fluid akin to human CSF that contains proteins and known molecular biomarkers in self-contained cystic structures (Pellegrini et al., 2020). The generation of these organoids from hiPSC lines can present an alternative source for patient-derived CSF samples, limiting the need for invasive procedures such as lumbar punctures to collect this fluid. There is evidence to suggest that ChP organoids at day 60 of development and beyond can demonstrate functionality of adult ChP, with the production of a mature hiPSC-derived *in vitro* cerebral spinal fluid (iCSF) by 100 days (Pellegrini et al., 2020). iCSF can be harvested at multiple timepoints to track changes of the presence and levels of proteins and inflammatory cytokines found within the iCSF, providing insight to the progression of disease. For example, mass spectrometry is a commonly used technique to perform intensive proteomic survey of human CSF (Macron et al., 2019; Núńez Galindo et al., 2019), and can be done on iCSF as well to further investigate the iCSF proteome of patients with rare neurological diseases. Proteomic data from iCSF samples can be compared to existing spectral libraries of human CSF (hCSF) proteomes from patients (Schilde et al., 2020), to confirm that iCSF is a valid alternative to hCSF for biomarker research and discovery. As shown in Figure 3, in addition to proteomic analysis, metabolomic (Yan et al., 2021) and lipidomic (Byeon et al., 2021) analyses can be performed on this fluid to provide a comprehensive characterization of the composition of patient-derived CSF, with the aim of detecting unique disease biomarker signatures compared to CSF from healthy patients. The signatures found using this *in vitro* model can also be integrated into generative computer modeling platforms to inform clinicians of patient disease progression, creating a translational bridge by turning these observations into informed patient interventions.

However, while hiPSC-derived neural organoid models provide a promising platform to study the molecular and cellular mechanisms underlying neurological diseases in human cells, they have their limitations. These models are isolated systems that generally do not give rise to cell types of non-neural lineages due to the promotion of neural induction during differentiation, have limited maturation of their existing cell types and possess an atypical physiology that cannot fully recapitulate the complex environment of the human brain (Andrews and Kriegstein, 2022). To address this limitation, researchers have been co-culturing 2D and 3D neural tissues with non-neural cell types (Figure 2A). Neural organoids also lack vascularization, limiting the nutrient and metabolite exchange from the innermost regions of these structures, resulting in cell death in those regions (Lancaster and Knoblich, 2014). In the case of ChP organoids, lack of vascularization also means that this system cannot reproduce one of the key functions of the ChP *in vivo*, the formation of the blood-cerebrospinal fluid barrier (B-CSF) (Solár et al., 2020). There are groups who have vascularized organoids (Solár et al., 2020), and while these approaches create space for passive media flow, it still doesn’t fully replicate blood flow. Despite these limitations, the ChP organoid model has the potential to provide a platform to allow further work on the identification and validation of biomarkers for rare neurodegenerative and neurodevelopmental diseases, with the aim of contributing to accurate and differential diagnoses and improved patient outcomes.

## 6 High-throughput approaches to disease-specific drug discovery

Clinical drug trials notoriously report poor outcomes, in part because drug discovery, development, and pre-clinical research have typically relied on non-mammalian model systems that lack important genetic and physiological features that are unique to the human brain. An important part of the challenge is that human primary cells are challenging to procure and propagate *in vitro*. With the emergence of hiPSCs and the ongoing collective efforts of the scientific community, directed differentiation methods can produce specific cell types which is enabling targeted pharmacological research into different neurodevelopmental and neurodegenerative diseases in which specific cell types are affected (i.e., dopaminergic neurons in PD, GABAergic neurons in Huntington’s disease) (HD iPSC Consortium, 2012; Doi et al., 2020). Also possible is the investigation of interactions between cell types [i.e., microglia and motor neurons in amyotrophic lateral sclerosis (ALS)] (Sances et al., 2016; Vahsen et al., 2022). Combined with CRISPR-Cas9 technology, the use of hiPSCs has revolutionized drug discovery by allowing the generation of *in vitro* models with much improved relevance to human brain development and cell type fidelity, and by providing a route to gain deeper understanding of the molecular mechanisms that drive human genetic diseases (Takahashi et al., 2007). In this regard, the ability to capture subcellular events in response to pharmacological interventions has been a boon to drug discovery.

High-throughput screening (HTS) is a technique to rapidly assay a range of variables in an automated way that inform cellular phenotypes in relation to genetic mutations or environmental conditions, and is particularly useful as a drug discovery tool. This application was driven by a growing need to find more effective and highly specific drug candidates in a diverse chemical space. In a typical HTS screen, small molecules are tested in parallel against biological targets (e.g., cells or simple tissues) to identify “hit” compounds that can restore normal phenotypes. HTS has been enabled by a confluence of several scientific and technological advances including: (1) the development of large, diverse, and individually characterized compound libraries; (2) improvements to cell-based assays that are cost-effective, involve straightforward protocols, and can probe intra- and inter-cellular events; (3) miniaturization of assays to reduce the cost-prohibitive nature of large screens; (4) advances in engineering of automation robotics to reduce repetitive manual tasks; and (5) development of bioinformatic tools for large dataset management and analysis (Broach and Thorner, 1996; Sittampalam et al., 1997; Fernandes, 1998; Silverman et al., 1998). Each of these fields remains highly active, and ongoing advancements continue to improve HTS and drive its widespread application. Here we will highlight selected areas that could advance drug discovery by harnessing the combined strength of hiPSCs and HTS.

One major advantage of hiPSCs is the continuous and unlimited generation of cells and tissues of interest. However, given the sheer volume of cells required for large screens, which can include 100,000 compounds or more, the manual labor required to culture hiPSCs and downstream cell types is not feasible for most laboratories. Though recently, innovations in automative engineering are addressing this issue by developing automated cell culture systems that entail strictly hands-off and scalable workflows. Liquid handlers and robotic arms can now perform tasks related to tissue culture vessel handling, media exchanges, subculturing, and differentiation protocols (Terstegge et al., 2007; Thomas et al., 2009; Valamehr et al., 2012; Conway et al., 2015; Tristan et al., 2021; Deng et al., 2023). Well-optimized automation can be reliably used to implement standard operating procedures, efficiently manage cell quality control, and reduce random batch-to-batch variability associated with human error. This is important because drug screens are costly assays, and as such it is common practice to have few or no technical or biological replicates. Thus, consistency of cellular and experimental parameters is a key concern as the few (or single) replicate measurements must accurately represent the phenotype(s) of interest.

Due to individual genetic diversity, effective precision medicine platforms will require patient-specific cell lines to appropriately classify both disease-specific cohorts and heterogeneity among patients with a given condition. Thus, scaling up automated cell culture technologies is critical to make this a reality. Efforts have been made to develop automated pipelines for the creation of hiPSC lines from fibroblasts (Paull et al., 2015). In the context of drug discovery, this will improve healthcare efficacy as top drug candidates can be identified before they are administered, thereby minimizing the deleterious side effects of medication optimization.

Despite the current advancements in hiPSC disease modeling, a unique challenge in neuroactive drug discovery using *in vitro* models is the absence of a BBB. The BBB is a layer of endothelial, mural, astrocytic, and immune cells surrounded by a basement membrane. It has a critical role in tightly regulating the transfer of substances between the circulatory system and the central nervous system, and serves to prevent exposure of the brain to harmful substances (Daneman and Prat, 2015). However, because the permeability of the BBB is very selective, most drug candidates are not effectively transported to the brain and only drugs below 400 Da are able to cross via lipid-mediated diffusion (Pardridge, 2012). Strategies have been developed that enhance the ability of drugs to penetrate the BBB, including increasing drug lipid solubility, use of carrier-mediated transport, or concurrent use of another drug that disrupts BBB permeability (Mikitsh and Chacko, 2014). Thus, consideration and inclusion of models to investigate permeability through the BBB is a critical aspect of *in vitro* drug discovery that needs to be increasingly considered when making predictions for drug efficacy.

Co-culture models that combine distinct hiPSC-derived cell types to replicate key aspects of the BBB exhibit physiologically appropriate characteristics including tube formation, organized tight junctions with acceptable transendothelial electrical resistance (TEER), expression of active transporters, and uptake of low-density lipoprotein (Lippmann et al., 2012; Aday et al., 2016; Qian et al., 2017). This stem-cell derived model, either as a standalone organoid or combined with organ-on-a-chip technology, shows great promise in enhancing drug discovery research by simulating a more physiologically accurate representation of drug delivery (Nzou et al., 2018; Choi et al., 2024). However, the low-volume nature of producing these models presents a challenge in translatability to HTS. Preliminary efforts have started scale up of the system through innovations in multi-chamber chip fabrication, which allows for parallel drug testing, but this technology has yet to be explored with HTS (Fan et al., 2016; Wevers et al., 2018).

Leveraging hiPSC technologies combined with HTS workflows is a cornerstone of translational medicine because it provides a powerful tool to elucidate disease pathways and identify potential pharmacological targets. However, limitations still exist. As described above, the benefits of organoid and assembloid modeling are evident, but incorporating these advances into a HTS workflow remains a barrier due to scalability limitations of 3D tissue models. Additionally, 3D models are typically heterogeneous in composition and size, which introduces high levels of error and thus are intrinsically challenging to characterize with currently available tools (Carragher et al., 2018; Booij et al., 2019). Additionally, compound hit identification relies primarily on a still limited range of fluorescent protein markers. This means that these assays have limited potential for multiplexity due to overlap in excitation and emission wavelengths. Thus, there is an ongoing demand for standardization of 3D tissue models (to produce tissues of reproducible size and architecture) and improved imaging probes, particularly for quantification of functional disease phenotypes and the short- and long-term intra- and inter-cellular responses with drug treatment.

Increasingly advanced hiPSC models have provided a greater understanding of disease pathogenesis, but to improve pharmacological interventions there exists a need for paradigm shifts in strategies for drug discovery. Historically, drug discoveries depended on observations of phenotypic changes of a single known target. To prevent off-target effects, pharmaceutical research aims to develop compounds with low promiscuity. However, this strategy has poor efficacy for complex diseases such as schizophrenia, multiple sclerosis (MS), and cancer. The problem arises because those diseases are polygenic or involve more complex interactions between different cell types or multiple molecular pathways. More recently, research has revealed the benefits of highly specific multi-target compounds in treating such multifactorial diseases, likely due to synergistic effects through secondary off-targets (Peters, 2013; Anighoro et al., 2014; Makhoba et al., 2020). Now coined as polypharmacology, this new approach to drug discovery aims to elucidate new applications of existing and theoretical compounds (Anighoro et al., 2014).

The forefront of drug discovery is moving toward incorporation of deep learning and artificial intelligence (AI) to aid in identifying drug candidates *in silico*. AI-assisted tools are continuously improving predictions and characterizations of cell protein folding and structure, identifying druggable proteomes, and simulating protein-ligand binding behavior (Cichońska et al., 2024). Combined with ultra-large virtual libraries, some with over 116 billion molecules that cover all possible structures within the chemical space, the combination of these powerful resources can generate an unprecedented amount of data for a targeted approach to polypharmacology (Ruddigkeit et al., 2012; Reymond, 2015). Well-characterized mechanisms of action can be invaluable for clinicians making critical treatment decisions and can greatly inform drug design and synthesis. Integration of AI-informed data with hiPSC platforms presents an exciting new frontier of disease modeling and pharmaceutical interventions. In parallel, the advance of high-throughput screening methods, empowered by the scalability of hiPSC-derived systems, holds the promise of expediting identification and validation of novel therapeutic compounds. Thus, with the right applications, hiPSCs are positioned to be particularly powerful clinical tools with outstanding patient-specific precision.

## 7 Integrating hiPSC-derived cellular data into AI-driven patient biophysical models

Early applications of AI toward hiPSC-derived models centered on image analyses to classify cells and hiPSC colonies based on morphological features, allowing for accurate classifications without human bias and enhancing precision and scalability by automating manual assessments (Vo et al., 2024). This laid the groundwork for the use of machine learning to automate the analysis of more complex data sets, such as RNA sequencing data, morphological and molecular assessment of disease-specific cell types, and cell function monitoring. In the context of disease-modeling, AI algorithms can become trained on large data sets to report alterations in cell morphology (Teles et al., 2021), fluorescent readouts of cell state and function (Nishino et al., 2021) to understand and identify differences in cell differentiation in hiPSCs, ultimately allowing it to predict differentiation outcomes. These predictions can be used to facilitate the high-throughput screening of drug candidates by analyzing cellular responses to treatments, providing insights to drug efficacy and toxicity (Kusumoto et al., 2022). Label-free drug screening systems can be especially useful in cases where no effective molecular markers for a given phenotype are known.

We envision that these achievements in AI around hiPSC-derived brain models can be further expanded to permit integration of meaningful cellular data with other types of patient-derived information that represent brain function or dysfunction on a larger scale. For example, rapid advancements in deep learning and generative AI have also led to the establishment of synthetic replicas of biophysical entities based on patient-derived neuroimaging data, coined “digital twins” (DT). In its most general sense, DT technology allows for the integration of large volumes of data to simulate a physical system in a digital space and to make predictive models. This concept has been harnessed for a myriad of applications spanning spacecraft navigation simulation (Grieves and Vickers, 2017) to urban planning (Bolton et al., 2018). In the context of biomedical research and precision medicine, there are promising new efforts that leverage the DT technology to integrate subject-derived data to form brain simulations, ultimately with the goal of using this predictive model to inform decision-making in patient diagnosis, prognosis, and therapeutics. For example, consolidation of longitudinal magnetic resonance imaging (MRI) to produce reference data on normal aging can be used to create an algorithm to identify premature development of brain atrophy and predict and monitor disease progression of MS (Voigt et al., 2021; Cen et al., 2023). Electroencephalography (EEG) theta and delta activity can be used as biomarker candidates to classify stroke patients to guide prevention and post-stroke care (Hussain and Park, 2021).

Previous work on a platform built on generative biophysical modeling, The Virtual Brain (TVB), has accurately generated high-fidelity patient brain models that can map trajectories for normative aging (Lavanga et al., 2023) and neurological conditions like neurodegeneration (Zimmermann et al., 2018) idiopathic epilepsy (Jirsa et al., 2014), stroke (Falcon et al., 2016) and cancer (Aerts et al., 2018). TVB has also shown that the excitatory/inhibitory (E/I) balance in different neuronal populations can provide a strong link between cellular and neuroimaging models (Deco et al., 2014; Proix et al., 2017; Schirner et al., 2018; Figure 1). This suggests that electrophysiological modeling can be used as a parameter to determine differences between patient and control iPSC-derived neural cultures. In the context of epilepsy disorders, changes in E/I balance can be used to determine the efficacy of drugs being tested on iPSC-derived neural tissue for treatment of the disease. As most patients have subtle differences in their disease-causing mutations, testing drugs on cultures derived from their own hiPSCs provides the most direct, translatable link if a drug candidate is identified. In further applications, the electrophysiology data collected from the aforementioned multi-region assembloid models allows for a detailed assessment of neural cell and circuit interactions between different brain regions, particularly as inhibitory interneurons integrate within excitatory neuron networks.

The integration of electrophysiological data from hiPSC-derived models into these AI-modeling platforms has the clinical potential to predict how cellular processes and their manipulation can impact the whole brain, providing estimates of brain function across multiple scales–individual cells, and single or interconnected neural networks–on an individualized basis. This integration can allow for the development of a fully personalized platform for rare diseases that can span population-level and patient-specific mechanisms, informing clinical strategies and cross-validated biomarkers. This approach can allow the rapid development of disease-specific diagnostics and individualized treatment plans for patients with rare neurological disorders.

In summary, as hiPSC-derived neural disease models evolve to more accurately reflect the complexity of human neurobiology *in vitro*, their potential to uncover disease mechanisms, identify biomarkers and therapeutic targets will only grow. The integration of hiPSC-derived neural models with advanced tools such as genome engineering, high-throughput screening and AI-modeling platforms offers a promising pathway toward the realization of precision medicine for brain disorders. Together, these innovations hold the promise of transforming the management of complex and rare neurological disorders to hopefully offer more effective and tailored therapeutic approaches.
